# Modulation of Designed Gut Bacterial Communities by Prebiotics and the Impact of Their Metabolites on Intestinal Cells

**DOI:** 10.3390/foods12234216

**Published:** 2023-11-22

**Authors:** Dalila Roupar, Abigail González, Joana T. Martins, Daniela A. Gonçalves, José A. Teixeira, Cláudia Botelho, Clarisse Nobre

**Affiliations:** 1 CEB—Centre of Biological Engineering, Campus de Gualtar, University of Minho, 4710-057 Braga, Portugal; dalila.roupar@ceb.uminho.pt (D.R.); alondra.colunga@ceb.uminho.pt (A.G.); joanamartins@deb.uminho.pt (J.T.M.); daniela.goncalves@ceb.uminho.pt (D.A.G.); jateixeira@deb.uminho.pt (J.A.T.); claudiabotelho@deb.uminho.pt (C.B.); 2 LABBELS—Associate Laboratory, 4710-057 Braga, Portugal

**Keywords:** fructo-oligosaccharides, *Aspergillus ibericus*, prebiotics, in vitro fermentation, gut microbiota, short-chain fatty acids, intestinal epithelium

## Abstract

The impact of prebiotics on human health is associated with their capacity to modulate microbiota, improving beneficial microbiota–host interactions. Herein, the prebiotic potential of microbial-fructo-oligosaccharides (microbial-FOSs) produced by a co-culture of *Aspergillus ibericus* plus *Saccharomyces cerevisiae* was evaluated on seven- and nine-strain bacterial consortia (7SC and 9SC, respectively), designed to represent the human gut microbiota. The 7SC was composed of *Bacteroides dorei*, *Bacteroides vulgatus*, *Bifidobacterium adolescentis*, *Bifidobacterium longum*, *Escherichia coli*, *Lactobacillus acidophilus*, and *Lactobacillus rhamnosus*. The 9SC also comprised the aforementioned bacteria, with the addition of *Bacteroides thetaiotaomicron* and *Roseburia faecis*. The effect of microbial-FOSs on the metabolic activity of intestinal Caco-2/HT29-MTX-E12 co-culture was also assessed. The results showed that microbial-FOS selectively promoted the growth of probiotic bacteria and completely suppressed the growth of *E. coli*. The microbial-FOSs promoted the highest production rates of lactate and total short-chain fatty acids (SCFA) as compared to the commercial prebiotic Frutalose^®^ OFP. Butyrate was only produced in the 9SC consortium, which included the *R. faecis*—a butyrate-producing bacteria. The inclusion of this bacteria plus another *Bacteroides* in the 9SC promoted a greater metabolic activity in the Caco-2/HT29-MTX-E12 co-culture. The microbial-FOSs showed potential as promising prebiotics as they selectively promote the growth of probiotic bacteria, producing high concentrations of SCFA, and stimulating the metabolic activity of gut cells.

## 1. Introduction

The gut microbiota is a complex microbial community that inhabits the human colon. It is mainly composed of Firmicutes, including bacteria from the genera *Lactobacillus*, *Bacillus*, *Clostridium*, *Enterococcus*, and *Ruminicoccus*, and of the Bacteroidetes, including *Bacteroides*, *Alistipes*, *Parabacteroides*, and *Prevotella* [1,2]. These dominant phyla represent 80 to 90% of the total microorganisms found in a healthy human gut microbiota [3]. On the other hand, only a small proportion of Actinobacteria (mainly represented by the *Bifidobacterium* genus), Proteobacteria, Fusobacteria, and Verrucomicrobia are present in the gut microbiota [4,5,6].

The gut microbiota plays an essential role in the maintenance of intestinal homeostasis and functionality [7]. Under normal conditions, the intestinal microbiota provides benefits to the host, such as energy harvesting, protection against pathogens, and immunomodulation [8]. This symbiotic relationship is impaired as a result of altered microbial composition, known as dysbiosis. Dysbiosis refers to changes in the microbiota composition usually characterized by a decrease (in number or type) in beneficial microorganisms, and an overgrowth of harmful ones [9]. Pathogenic microorganisms promote the release of proinflammatory cytokines, which damage intestinal tight junctions (TJs) leading to disruption of the intestinal barrier, commonly known as the “leaky gut” [7]. The intestinal barrier damage results in intestinal and systemic inflammation which plays an important role in the pathogenesis of multiple intestinal conditions (e.g., irritable bowel syndrome, Crohn’s disease) as well as extraintestinal conditions (e.g., diabetes, obesity, atopic dermatitis) [10,11,12,13]. Therefore, the modulation of gut microbiota composition and diversity through the use of probiotics and prebiotics is considered a promising method to overcome dysbiosis.

Prebiotics are fermented food ingredients that enable specific changes in the composition and activity of the gut microbiota for the benefit of the host’s health [14]. Therefore, prebiotics must be resistant to gastrointestinal digestion, reaching the large intestine and the caecum intact for fermentation by the microbiota. Currently, fructans are the most popular prebiotics, which include inulin and fructo-oligosaccharides (FOSs) [15]. FOSs are recognized as prebiotics and have been shown to promote a positive effect on gut microbiota composition and metabolic activity [15,16]. FOS are present in many fruits and vegetables, but may also be produced by microbial fermentation (microbial-FOSs) [15]. FOS consumption by probiotic bacteria can strategically modulate the gut microbiota, contributing to (i) an increased short-chain fatty acids (SCFAs) production, (ii) a pH reduction of the luminal colon, (iii) a suppression of enteric pathogens growth, and (iv) a potential indirect barrier-preserving mechanism [7,17].

In particular, the *Lactobacillus* and *Bifidobacterium* genera stand out for their probiotic activity and susceptibility to the prebiotic action [6]. They have been associated with individuals’ well-being and immune system maintenance [5]. SCFAs are the main products resulting from prebiotic fermentation by this probiotic microbiota. SCFAs are known to provide energy to the colonocytes, enhancing intestinal barrier function, helping to repair wounded epithelium, and acting as postbiotic molecules [18,19,20]. The main SCFAs produced are acetate, propionate, and butyrate, which comprise approximately 95% of all SCFAs produced by the human gut microbiota [21], whereas only a small amount of branched-chain fatty acids (BCFA, isobutyrate, valerate, and isovalerate) and organic acids (lactate, succinate, formate) are formed [22,23]. The acetate–propionate–butyrate proportion in the human gut is approximately 3:1:1, respectively. Nonetheless, this proportion depends on the substrate available, initial microbiota composition, and gut transit time [21]. Despite all the evidence of the beneficial effects of SCFAs on gut microbiota balance and epithelial barrier integrity, the effect of prebiotics on the stability of larger microbiota consortia and intestinal epithelial cells’ metabolism is yet to be fully unveiled [24].

Therefore, this study aimed to investigate the potential changes in microbial-derived metabolites in response to different prebiotics in an in vitro fermentation system. In this experiment, seven or nine bacterial strains, representative of the gut microbiota (7SC and 9SC, respectively), were used. The 7SC consortium consisted of *Bacteroides dorei*, *Bacteroides vulgatus*, *Bifidobacterium adolescentis*, *Bifidobacterium longum*, *Escherichia coli*, *Lactobacillus acidophilus*, and *Lactobacillus rhamnosus*. The 9SC consortium included the aforementioned bacteria, along with the addition of *Bacteroides thetaiotaomicron* and *Roseburia faecis*. A microbial-FOS produced in previous work by a co-culture of *Aspergillus ibericus* with *Saccharomyces cerevisiae* YIL162 W [25] and a commercial non-microbial FOS (Frutalose^®^ OFP), were selected to conduct this work. Furthermore, the effect of the generated microbiota metabolites on the metabolic activity of intestinal Caco-2/HT29-MTX-E12 co-culture was also assessed.

## 2. Materials and Methods

### 2.1. Carbohydrates Tested

Three different carbohydrates were tested as the main substrates for bacterial fermentation: (i) microbial-FOS, a microbial fructan produced in our lab; (ii) Frutalose^®^ OFP, a non-microbial commercial fructan gently offered by Sensus (Roosendaal, The Netherlands); and (iii) glucose, a non-prebiotic carbohydrate purchased at VWR (Radnor, PA, USA).

The microbial-FOS samples were produced using an integrated fermentation strategy. Briefly, *Aspergillus ibericus* MUM 03.49, from Micoteca of University of Minho culture collection (MUM, Braga, Portugal) was co-cultured with *Saccharomyces cerevisiae* YIL162 W (EUROSCARF) for simultaneous FOS production and purification. The microbial-FOS mixture obtained was desalted with activated charcoal using the process described in Roupar et al., 2022 [26]. Briefly, 40 mL of fermentative broth mixture was treated with 10 g of activated charcoal for 3 h at 165 rpm and 25 °C. Next, small sugars were removed by washing the activated charcoal three times with 150 mL of ultra-pure water. FOSs were desorbed with 100 mL of ethanol (50% *v*/*v*) for 1 h, at 165 rpm and 25 °C. After a filtration step, samples were evaporated at 60 °C using a rotary evaporator (B. Braun Biotech International) to remove the ethanol and concentrate the carbohydrates before freeze drying (Heidolph lyophilizer).

### 2.2. Microbial Community

*Bacteroides dorei* (DSM 17855), *Bacteroides vulgatus* (DSM 1447), *Bacteroides thetaiotaomicron* (DSM 2079), *Bifidobacterium longum* (DSM 20219) and *Roseburia faecis* (DSM 16840) were acquired from DSMZ GmbH (Braunschweig, Germany). *Bifidobacterium adolescentis* (CECT 5781), *Escherichia coli* (CECT 736), *Lactobacillus acidophilus* (CECT 288), and *Lactobacillus rhamnosus* (ATCC 4356) were obtained from the MUM culture collection (Braga, Portugal).

Two defined bacterial consortia were tested. The first consortium (7SC) was composed of 7 bacteria: *B. dorei*, *B. vulgatus*, *B. adolescentis*, *B. longum*, *E. coli*, *L. acidophilus*, and *L. rhamnosus*. The second consortium (9SC) included all the bacteria of the 7SC plus *B. thetaiotaomicron* and *R. faecis*. Aliquots of each mentioned bacterium were preserved in its respective growth culture medium (Table 1) with the addition of 25% glycerol, at −80 °C.

Firstly, bacteria were inoculated at 10% *v*/*v* in 10 mL of their respective media (Table 1) under anaerobic conditions (80:20 N_2_:CO_2_ at 2 bar), at 37 °C, and for 24 h. Further, bacteria were re-inoculated at 10% *v*/*v* in 10 mL fresh media for another 24 h, under the same conditions. The grown bacteria were used as inoculum in the fermentation experiments.

### 2.3. Fermentation Assays

Fermentations were run in a culture medium, herein named as FEED, with the following composition: arabinogalactan (1 g·L^−1^), corn starch (4 g·L^−1^), mucin-type III (4 g·L^−1^), yeast extract (4 g·L^−1^), xylan (1 g·L^−1^), peptone (5 g·L^−1^), guar gum (1 g·L^−1^), tryptone (4 g·L^−1^), Tween 80 (1 mL·L^−1^), L-cysteine (0.5 g·L^−1^), hemin (0.01 g·L^−1^), vitamin B12 (0.005 g·L^−1^), and an electrolyte solution (10 mL·L^−1^) containing NaCl (4.5 g·L^−1^), NH_4_Cl (0.4 g·L^−1^), KCl (0.25 g·L^−1^), KH_2_PO_4_ (0.4 g·L^−1^), and MgCl·6H_2_O (0.15 g·L^−1^) [27].

An amount of 20 g·L^−1^ of the carbohydrate being tested, namely microbial-FOS, Frutalose^®^ OFP, or glucose (positive control), was added to the FEED media. A negative control (i.e., FEED medium with no-added carbohydrates) was also prepared.

Briefly, 60 mL of each medium (in triplicate) was added to glass anaerobic culture bottles (80:20 N_2_:CO_2_), which were further autoclaved at 121 °C for 20 min. The FEED medium was further inoculated (10% *v*/*v*) with the 7SC or 9SC consortium. Equal volumes of each bacterial strain were used as the inoculum. Fermentations were run for 30 h at 37 °C. Samples were harvested at 0, 6, 12, 24, and 30 h of fermentation for optical density (OD) analysis at 600 nm, pH measurements, viable bacterial count (Section 3.1), and analysis of sugars and organic acids using high-performance liquid chromatography (HPLC) (Section 3.2 and Section 3.3).

Arabinogalactan, xylan, and L-cysteine were acquired from TCI (Tokyo, Japan); guar gum, mucin type III, vitamin B12, NH_4_Cl and MgCl·6H_2_O from Sigma-Aldrich (St. Louis, MO, USA); corn starch and Tween 80 from Riedel de Haën (Charlotte, NC, USA); NaCl, KCl and KH_2_PO_4_ from Panreac AppliChem (Barcelona, Spain); bacteriological peptone and tryptone from Oxoid (Basingstoke, UK); and yeast extract from Leofilchem (Roseto degli Abruzzi, Italy).

#### 2.3.1. Bacterial Growth Analysis

Bacterial growth was analyzed using viable bacterial counts after 30 h of fermentation at 37 °C. Four selective plating agar media were used: Wilkins–Chalgren anaerobic agar base (Himedia, India) supplemented with 0.01 g·L^−1^ Hemin (Sigma-Aldrich) for Bacteroides spp. [28]; Rogosa agar (Himedia, India) for *Lactobacillus* spp. [29]; MacConkey agar (Himedia, India) for *Enterobacteriaceae* [29]; and Bifidobacterium agar modified (Himedia, India) for *Bifidobacterium* spp. [30].

Samples were serially diluted and plated (0.05 mL) on each agar medium. Plates were then incubated in anaerobic jars for 48 h at 37 °C.

#### 2.3.2. Carbohydrates and Organic Acids Analysis

Carbohydrates consumption, SCFAs (i.e., acetate, propionate, and butyrate), and lactic acid production during fermentation were identified and quantified using HPLC. The fermentative samples were centrifuged at 8000× *g* for 10 min, and the obtained supernatants were filtered with acetate cellulose syringe filters with a cut-off of 0.22 μm (Orange Scientific, Braine-l’Alleud, Belgium) prior to analysis with HPLC.

Carbohydrates analysis was performed using an HPLC system (Jasco, Tokyo, Japan), equipped with an Asahipak NH2P-50 4E column (5 μm; 25 cm (length) × 0.46 cm (diameter), Shodex, Japan) connected to an Asahipak NH2P-50G 4A pre-column (4.6 mm (length) × 10 mm (diameter), Shodex, Japan), and a refractive index detector. Samples (20 μL) were eluted with a mixture of 70:30 *v*/*v* acetonitrile (HPLC Grade, Fisher Scientific, Waltham, MA, USA) and ultra-pure water, including 0.04% of ammonium hydroxide in water (HPLC Grade, Sigma-Aldrich, St. Louis, MO, USA). The elution was conducted at a flow rate of 1 mL·min^−1^ and 30 °C [26]. The chromatographic signal was recorded and further integrated using the Star Chromatography Workstation 6.3 software (Varian, CA, USA). The FOS standards used were purchased from FUJIFILM Wako Chemicals (Tokyo, Japan), namely, GF_2_: 1-kestose, GF_3_: nystose, and GF_4_: 1^F^-1-β-d-fructofuranosylnystose, and sucrose, fructose, and glucose standards were acquired from VWR (Radnor, PA, USA).

SCFA and lactic acid analyses were performed using a HPLC system equipped with an Aminex HPX-87H chromatographic column (8 µm; 30 cm × 0.46 cm length × diameter) from Bio-Rad (Hercules, CA, USA) and an ultraviolet spectrophotometer detector. Detection was performed at 210 nm. Samples (20 μL) were eluted in a sulfuric acid solution (5 mM) at a flow rate of 0.6 mL·min^−1^. The column temperature was set at 60 °C [26]. The chromatographic signal was recorded and further integrated using the Star Chromatography Workstation 6.3 software (Varian, CA, USA). SCFA and lactic acid standards were obtained from Merck (Darmstadt, Germany).

### 2.4. Cell Culture Experiments

Caucasian colon adenocarcinoma cells, Caco-2 from American Type Culture Collection (Washington, DC, USA) (ATCC^®^HTB37™) and HT29-MTX-E12 cells from European Collection of Authenticated Cell Cultures (Sailsbury, UK) (ECACC 12040401), were seeded in T-25 (25 cm^2^) flasks. Cell cultures were maintained at 37 °C in a humidified atmosphere with 5% CO_2_. Dulbecco’s Modified Minimum Essential Medium (DMEM) (Biowest, Nuaillé, France) enriched with non-essential amino acids (1%), penicillin–streptomycin–amphotercin (1%), both purchased from PAN-Biotech (Aidenbach, Germany), and 15% heat-inactivated fetal bovine serum (FBS) obtained from Merck (Darmstadt, Germany), were used to maintain cells growth until confluence. FBS content was then reduced to 10% when cell culture acquired confluence. Media was changed every second day. The integrity of the monolayer and the presence of domes were monitored regularly by phase-contrast microscopy. Cell cultures were used for experiments after 14 days post-seeding.

#### Cell Metabolic Activity Assay

Cell metabolic activity was assessed using resazurin reduction assay [31]. Briefly, Caco-2/HT29-MTX-E12 co-cultures (at 9:1 ratio) were seeded at 1 × 10^5^ cells·cm^−2^ in 48-well plates and grown for 14 days to form a differentiated monolayer with mucus formation. Then, the cell culture medium was removed and replaced with the fermentation metabolites (previously filtered through 0.22 μm pore size, to remove bacteria), diluted in order to obtain various concentrations in the culture medium (i.e., 1:3, 1:7, and 1:11), and incubated for 24 h, at 37 °C, and 5% CO_2_. After incubation, the medium was removed, and the cells were carefully washed with phosphate-buffered saline (PBS) purchased from Lonza (Basel, Switzerland). Then, 400 µL of DMEM containing resazurin (Sigma-Aldrich, St. Louis, MO, USA) at 0.5 mM (previously prepared in PBS) was added to each well and incubated for 2 h at 37 °C. The supernatant (100 µL) of each well was placed on 96-well black microplate, and fluorescence of the reduced resorufin product was measured at λ_ex_ = 560 nm and λ_em_ = 590 nm using a microplate reader (Cytation 3, BioTek Instruments, Inc., Winooski, VT, USA). The experiments were conducted at least in triplicate for each sample and dose. The results were expressed as a cell metabolic activity percentage in relation to the control (i.e., non-treated cells) using the following equation: (Fluorescence intensity of treated cells/Fluorescence intensity of the control) × 100.

### 2.5. Statistical Analysis

The statistical analysis was carried out using GraphPad Prism 9.5.1 (San Diego, CA, USA). The normality of the data’s distribution was evaluated through Shapiro–Wilk’s test. As the data proved to follow a normal distribution, the one-way ANOVA coupled with Tukey’s post hoc test or the student’s *t*-test was used to determine significant differences in the evolution of the bacteria growth and their metabolic activity, at each time point. Repeated Measures ANOVA was used to evaluate the bacterial population over time. Differences were considered significant for *p*-values ≤ 0.05.

## 3. Results

### 3.1. Bacterial Growth and pH Profile

**Figure 1:**
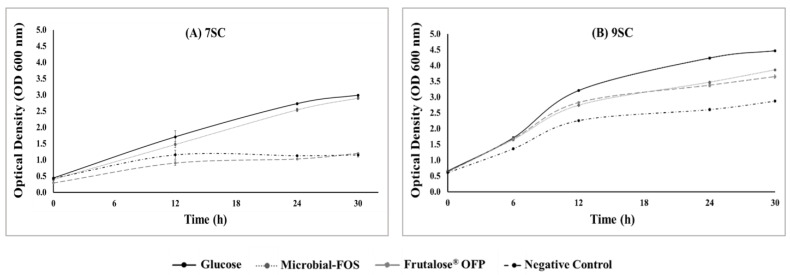
Growth curves of in vitro batch fermentations (**A**) of the seven-strain community (7SC) and (**B**) of the nine-strain community (9SC).

Regarding bacterial growth, both consortia (7SC and 9SC) were able to grow in FEED medium under anaerobic conditions, regardless of the substrate used (Figure 1A,B). Regarding OD curves obtained for each substrate, it is possible to observe that bacteria grew quicker and with a higher density in the following order: glucose > microbial-FOS > Frutalose^®^ OFP > negative control. As shown in Figure 1A, glucose and microbial-FOS were the substrates that better stimulated 7SC bacterial growth during fermentation (*p* < 0.05), achieving OD values of 2.99 ± 0.02 and 2.90 ± 0.04 (*p* = 0.187), respectively, after 30 h fermentation. FEED medium with Frutalose^®^ OFP (OD 1.20 ± 0.01) attained a microbial growth similar to the negative control (OD 1.15 ± 0.06) since no significant statistical differences were observed between both values (*p* = 0.655). The OD values obtained with the 9SC (glucose: 4.47 ± 0.02; microbial-FOS: 3.86 ± 0.02, Frutalose^®^ OFP: 3.65 ± 0.05, and negative control: 2.87 ± 0.03) (Figure 1B) were higher than the ones obtained for the 7SC (*p* < 0.001), indicating a significantly higher bacterial growth of the 9SC than the 7SC in all tested substrates.

**Figure 2:**
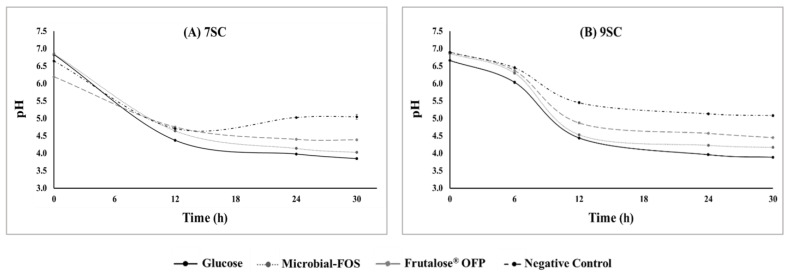
Variation of pH during in vitro fermentations with (**A**) seven-strain consortia (7SC) and (**B**) nine-strain consortia (9SC).

Figure 2A,B show the pH changes throughout batch fermentations. The initial pH of all fermentation media after inoculation was around 6.5. In the first 12 h fermentation, the pH decreased significantly for all experiments (*p* < 0.05), which is coincident with the exponential bacterial growth phase observed in the OD results (Figure 1A,B). After 12 h, the pH values of the 7SC and 9SC fermentations continued to decrease, with the exception of the negative control in 7SC fermentation, in which a slight increase in the pH value was noticeable. Bacterial growth was reduced on the negative control after 12 h fermentation, presumably due to the depletion of the limited carbon source of this fermentation medium. The lowest pH values were observed for fermentations run with glucose and microbial-FOS as carbon sources: 3.85 ± 0.02 and 4.03 ± 0.01, respectively, for 7SC, and 3.89 ± 0.01 and 4.17 ± 0.01, respectively, for 9SC. On the other hand, the pH values decreased slightly during fermentation for the negative control, achieving pH values of 5.05 ± 0.07 and 5.08 ± 0.01 for 7SC and 9SC, respectively. Significant differences in pH values were found between the 7SC and 9SC fermentation for each tested substrate (*p* < 0.05), with the exception of the negative control (*p* = 0.254).

**Figure 3:**
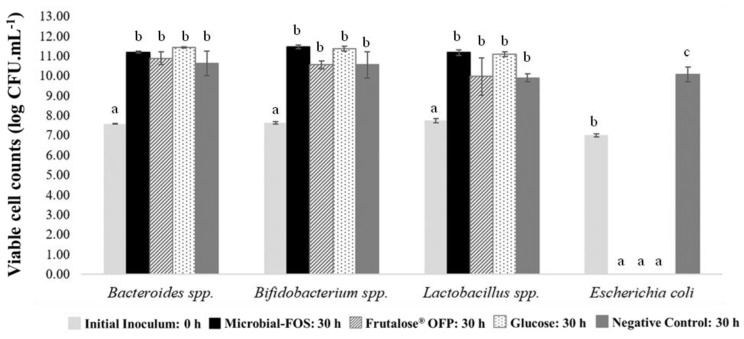
Viable cell counts of *Bacteroides* spp., *Bifidobacterium* spp., *Lactobacillus* spp., and *Escherichia coli* after 30 h fermentation with seven-strain consortia (7SC), with microbial-fructo-oligosaccharides (microbial-FOS), Frutalose^®^ OFP, glucose, and without additional carbohydrate (negative control) and incubated for 48 h at 37 °C. Values are expressed as log CFU·mL^−1^ ± SD. Different superscript letters correspond to significantly different viable cell counts (*p* < 0.05).

The specific growth of the *Bacteroides* spp., *Lactobacillus* spp., and *Bifidobacterium* spp. included in the 7SC was promoted by all tested carbon sources, without significant statistical difference between them, while the growth *E. coli* was suppressed during fermentations run with all the carbon sources tested, except for the negative control (Figure 3).

### 3.2. Carbohydrate Consumption

**Figure 4:**
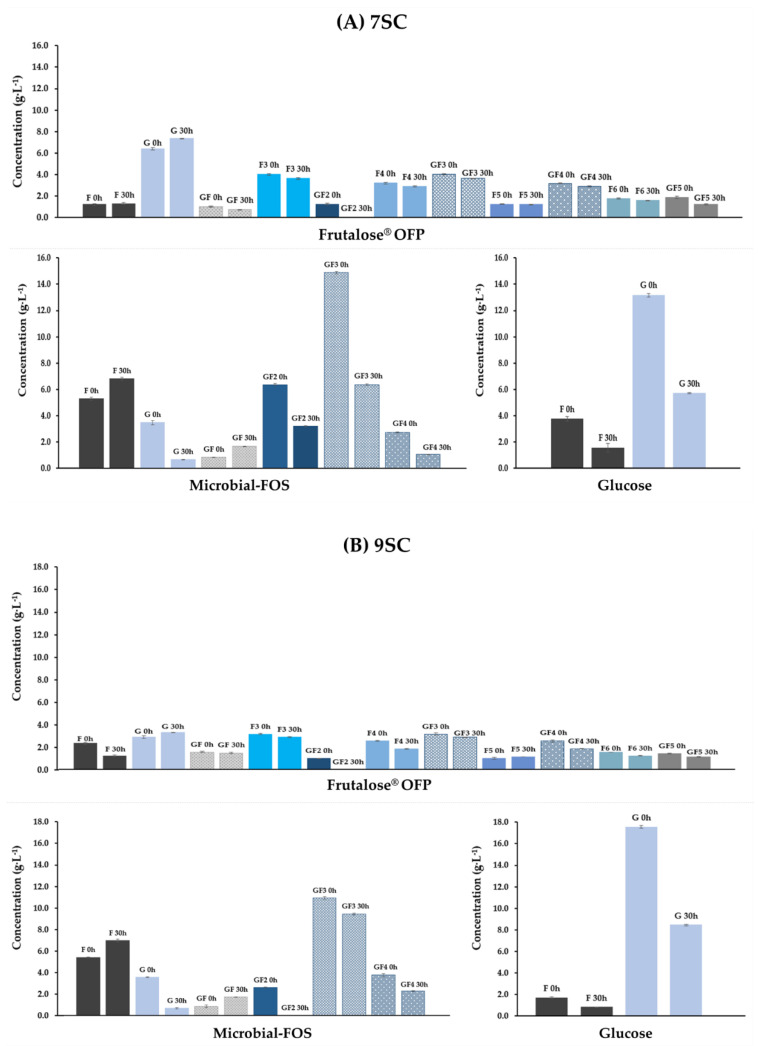
Carbohydrate profiles before and after 30 h fermentation with the bacterial consortium (**A**) 7SC and (**B**) 9SC. Carbohydrates: microbial-FOS: microbial-fructo-oligosaccharides, Frutalose^®^ OFP, F: fructose, G: glucose, GF_2_: 1-kestose, GF_3_: nystose, GF_4_: 1^F^-1-β-d-fructofuranosylnystose, F_n_ (2 < *n* < 6): inulin-type.

**Figure 5:**
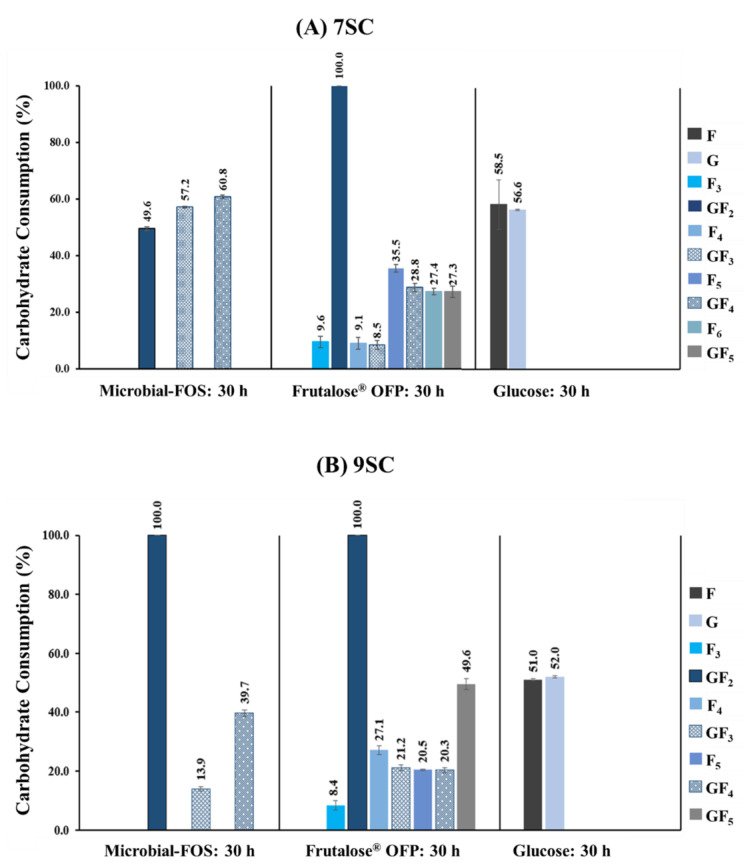
Profile of the carbohydrate consumption after 30 h fermentation with the bacterial consortium (**A**) 7SC and (**B**) 9SC. Carbohydrates: microbial-FOS: microbial-fructo-oligosaccharides, Frutalose^®^ OFP, F: fructose, G: glucose, GF_2_: 1-kestose, GF_3_: nystose, GF_4_: 1^F^-1-β-d-fructofuranosylnystose, F_n_ (2 < *n* < 6): inulin-type.

Figure 4A,B illustrate the concentration profile of each sugar present in the microbial-FOS, Frutalose^®^ OFP, and glucose samples before inoculation (0 h) and after 30 h of fermentation with the two defined bacterial consortiums (7SC and 9SC). Figure 5A,B show the carbohydrate consumption profiles. All carbohydrates were extensively metabolized by the bacterial communities, but their consumption pattern differed.

At the beginning of batch fermentations with 7SC, the microbial-FOS mixture contained in the FEED medium had a content of 24.34 ± 0.21 g·L^−1^ oligosaccharides and 9.78 ± 0.32 g·L^−1^ smaller carbohydrates, while Frutalose^®^ OFP held 20.40 ± 0.42 g·L^−1^ oligosaccharides and 8.64 ± 0.23 g·L^−1^ smaller carbohydrates. After 30 h of fermentation, the concentration of oligosaccharides in the microbial-FOS decreased to 10.82 ± 0.08 g·L^−1^ and in Frutalose^®^ OFP to 15.33 ± 0.23 g·L^−1^, representing a metabolization of 55.57 ± 0.31% and 24.82 ± 1.15% oligosaccharides, respectively. The glucose carbon source was consumed at a rate of 56.6 ± 0.4% by these consortia strains.

Regarding the 9SC fermentations, the initial composition of microbial-FOS in the FEED medium was 17.18 ± 0.28 g·L^−^^1^ oligosaccharides and 5.41 ± 0.24 g·L^−^^1^ smaller carbohydrates, while Frutalose^®^ OFP contained 15.18 ± 0.47 g·L^−^^1^ and 6.69 ± 0.29 g·L^−^^1^, respectively. After 30 h of fermentation, the content of oligosaccharides in the microbial-FOS decreased to 11.60 ± 0.09 g·L^−^^1^ and in Frutalose^®^ OFP to 11.19 ± 0.12 g·L^−^^1^, suggesting a metabolization of 32.48 ± 0.52% and 26.37 ± 0.76% oligosaccharides, respectively.

Concerning the initial carbohydrate concentration ratios in the 7SC fermentations, a GF_2_:GF_3_:GF_4_ ratio of 2.3:5.4:1.0 was observed for the microbial-FOS and a GF_2_:GF_3_:GF_4_:GF_5_ ratio of 1.0:1.42:3.0:2.6 was observed for Frutalose^®^ OFP. However, in the 9SC fermentations, the microbial-FOS had a GF_2_:GF_3_:GF_4_ ratio of 1.0:4.2:1.4 and Frutalose^®^ OFP had a GF_2_:GF_3_:GF_4_:GF_5_ ratio of 1.0:1.6:3.0:2.7.

The 7SC seems to preferentially consume microbial-FOS, presenting a higher DP: 60.8 ± 0.6% of GF_4_ (1^F^-1-β-d-fructofuranosylnystose), 57.2 ± 0.3% of GF_3_ (nystose) and 49.6 ± 0.7% GF_2_ (1-kestose) (Figure 5A). Regarding Frutalose^®^ OFP, 7SC completely consumed the GF_2_ and at least 8% of the F_3_ (inulotriose), F_4_ (inulotetraose), and GF_3_, and 27% of the F_5_ (inulopentaose), GF_4_, F_6_ (inulohexaose), and GF_5_ (1^F^-(1-β-d-fructofuranosyl)2-nystose) (Figure 5A).

Regarding the bacterial consortium with nine microorganisms, it appears to preferentially consume GF_2_, which was completely metabolized, followed by 39.7 ± 1.1% of GF_4_, and lastly by 13.9 ± 0.8% of GF_3_ (Figure 5B). In the batch fermentations with Frutalose^®^ OFP, GF_2_ was completely metabolized, followed by GF_5_ (49.6 ± 1.7%) and at least 20% of the other carbohydrates present in the Frutalose^®^ OFP sample (Figure 5B). The glucose consumption rate was 52.0 ± 0.4%.

### 3.3. Short-Chain Fatty Acid Production

The effect of several prebiotic compounds on the 7SC and 9SC was assessed by measuring the changes in total and individual content of SCFAs during the in vitro batch fermentations, namely acetate, propionate, and butyrate. Lactate, a major organic acid produced from the fermentation of non-digestible carbohydrates [32], was also investigated (Table 2). It is worth mentioning that the original fermentation medium composition already contained SCFAs, and the medium used for 9SC presented a higher SCFA content than the original fermentation medium because R. faecis growth medium contains a volatile fatty acid mixture.

During fermentation, both consortia (7SC and 9SC) were able to produce acetate, propionate, butyrate, and lactate, regardless of the substrate tested. The highest total SCFA concentration obtained in 7SC fermentation was observed in the medium that was rich in glucose (3.91 ± 0.07 g·L^−^^1^) after 12 h fermentation, as well as in microbial-FOS (4.11 ± 0.04 g·L^−^^1^) after 30 h, as compared to all the other samples (*p* < 0.05). However, in 9SC fermentation, the highest total SCFA content was obtained with microbial-FOS as the substrate, standing out from the other substrates (*p* < 0.05) (7.60 ± 0.23 g·L^−^^1^ and 7.83 ± 0.20 g·L^−^^1^, at 12 h and 30 h, respectively).

The SCFA production pattern was different for each bacterial consortium (Table 2). The highest amount of acetate was produced by 7SC and 9SC when the microbial-FOS and Frutalose^®^ OFP were used as the substrate, respectively (*p* < 0.001). The results also showed that the 9SC fermentation samples with glucose and Frutalose^®^ OFP had a higher acetate concentration as compared to the acetate production obtained in the 7SC fermentation; however, this difference was only statistically significant in the Frutalose^®^ OFP treatment (*p* < 0.001) (Table 2).

Regarding propionate production, the highest propionate concentration was observed for 7SC fermentations with glucose (*p* < 0.001) as a substrate at 12 h and 30 h (Table 2). However, a higher propionate concentration was obtained for 9SC fermentation samples using microbial-FOS and glucose than the other carbohydrate sources (*p* < 0.001), at 12 h and 30 h. It was also found that the highest levels of propionate concentration were obtained from the 9SC fermentation after 12 h and 30 h (*p* < 0.05) compared to all treatments.

According to the results shown in Table 2, butyrate was produced by the 9SC, which was an expected result since the main butyrate producing-bacteria in the human gut belong to the phylum *Firmicutes*, in particular *Faecalibacterium prausnitzii*, *Clostridium leptum*, *Eubacterium rectale*, and *Roseburia* spp. [33]. A strong butyrogenic effect was observed in microbial-FOS-added sample (*p* < 0.001), as butyrate production was higher than the negative control, glucose-added, and Frutalose^®^ OFP-added media, at 12 h and 30 h.

Lactate concentration increased over the entire fermentation time, and it was the main organic acid produced for all bacterial consortia treatments (Table 2). The glucose-added medium in the 7SC fermentation (at 12 h) showed higher lactate content (*p* < 0.001) than the other carbohydrate-added media, while the microbial-FOS-added samples had the highest lactate levels (*p* < 0.001) out of all the carbohydrate-added media at the end of the fermentation. Regarding 9SC fermentation, a higher lactate concentration was observed on Frutalose^®^ OFP samples (*p* < 0.001) at 12 h compared to other samples. However, the glucose-added samples presented the highest lactate concentration (*p* < 0.001) compared to all samples, after 30 h (Table 2).

### 3.4. Effect of Metabolites Produced by Different Microbial Communities Using Distinct Carbon Sources on an Intestinal Cell Co-Culture

The importance of the metabolites produced by the intestinal microbiota on different metabolic pathways has been widely described in the literature: from the inflammatory cascade to carcinogenic metabolism, as well as cell proliferation, differentiation, and apoptosis [34,35,36].

**Figure 6:**
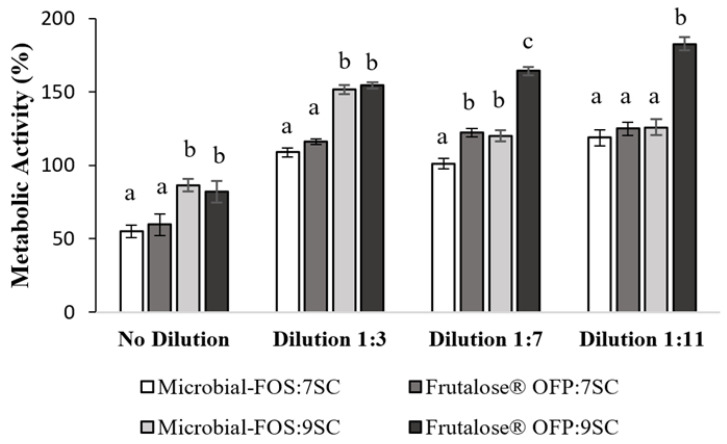
Metabolic activity of Caco-2/HT29-MTX cells co-culture upon 24 h exposure to metabolites at volume ratios of 1:3, 1:7, and 1:11, resulting from two bacterial consortia (7SC and 9 SC) fermentation using different carbon sources (microbial-FOS and Frutalose^®^ OFP). Different superscript letters correspond to significantly different cellular metabolic activity (*p* < 0.05).

Figure 6 describes the cellular metabolic behavior of an in vitro intestinal cell co-culture (Caco 2/HT29-MTX-E12) model used to evaluate the effect of the complex metabolites’ mixture produced in the presence of a potential prebiotic (microbial-FOS), and a commercial prebiotic (Frutalose^®^ OFP) by the 7SC and 9SC for 24 h. All metabolite samples at maximum concentration (i.e., non-diluted samples) induced a decrease in the metabolic activity of the intestinal cells. However, the resazurin reduction assay showed that the cell metabolic activity was higher than 100% for all the diluted fermentation metabolites tested. In addition, no significant metabolic activity differences were observed (*p* > 0.05) between the carbon sources used by the 7SC or 9SC. On the other hand, the metabolites produced by the 9SC presented a significant increase (*p* < 0.05) on the metabolic activity in comparison to the metabolites produced by 7SC.

## 4. Discussion

As shown in Figure 1 and Figure 2, the bacterial growth and the reduction in pH were particularly noticeable in the first 12 h of fermentation. After that time, the bacterial growth stopped for the negative control and became slower for the rest of the treatments, and the pH slowly continued to reduce for all the tested carbon sources. The decrease in pH value is a result of the release of SCFAs and other acidic metabolites produced during the fermentation [37]. Meanwhile, there was a slight pH increase for the negative control. Once the carbon source becomes limited, metabolite production and bacterial growth decrease, and therefore, pH increases, which is known as positive feedback [38]. On the other hand, bacteria from the 9SC continued to slowly grow for an extended period, even having the same carbon source. It is possible that this result is due to different cross-feeding interactions within the microbial community, resulting in less microbial competition for the initial substrate.

As expected, microbial-FOS had a similar effect to commercial FOS in promoting probiotic bacterial growth. In a previous work, the microbial-FOS produced by the *A. ibericus* (without the presence of the *S. cerevisiae*) has already been shown to promote the growth of *Bifidobacteria* and *Lactobacillus* species when cultured individually [39]. The bifidogenic effect of the microbial-FOS (produced in co-culture with *S. cerevisiae*) was also observed in fermentations run with human microbiota [26]. Both microbial-FOS and Frutalose^®^ OFP comprise a mixture of fructans of different degrees of polymerization (DP): DP 2-7 and DP 2-10, respectively. In a study conducted in vivo where fructans with different DP were administered to rats, it was found that lactobacilli were mainly stimulated by fructans presenting DP 4, DP 8, and DP 16, while bifidobacteria preferred DP 8, DP 16, and DP 23 oligosaccharides [40]. Nevertheless, the selective utilization of substrates was related to the species of bacteria rather than to the genus. As a matter of fact, *L. acidophilus* and *B. thetaiotaomicron* have been reported to be able to consume GF_2_ and GF_3_, while *L. rhamnosus* only used GF_2_ [41]. However, the processes of substrates’ degradation and consumption are not linear when working with a microbial community, as it is with a pure culture. In community, there are cross-feeding reactions between bacteria and the complexity of the medium, which also contains other sources of nutrients that can be used by the different bacteria [41].

*Lactobacilli* and *Bifidobacteria* are the most widely studied probiotic bacterial genera. The abundance of *Bifidobacteria* in the human gut is associated with a healthy state, while its deficiency has been linked to several diseases. Besides helping to degrade indigestible carbohydrates, *Bifidobacteria* contribute to the biosynthesis of vitamin B9 and tryptophan-derived indoles, which potentially improve gut barrier functionality and the immune response on the mucus layer [42]. Moreover, *Lactobacilli* produce bacteriocins and antimicrobial peptides that inhibit pathogen growth and improve the host’s immune system function [43]. In this context, the suppression of *E. coli*, an opportunistic pathogenic bacterium, might be a result of bacteriocin release. Additionally, the reduction in pH could be attributed to lactate production by *Lactobacillus* ssp. and SCFA produced by the bacterial consortia. It has been reported that the growth of *E. coli* is inhibited under acidic conditions (typically below 5.5) [44]. Additionally, it has been observed that a diet low in carbohydrates that are fermentable by intestinal bacteria can foster the proliferation of potential enteropathogenic microorganisms. This may also lead to an increase in proteolysis, and consequently, the generation of undesirable metabolites [45,46]. Such conditions could potentially explain the proliferation of *E. coli* in the negative control.

Microbial-FOSs consist of fructosyl moieties linked to a starting d-glucose unit (GF*_n_*-type, 2 < *n* < 4), since they are enzymatically synthesized from sucrose, while Frutalose^®^ OFP is a mixture of F*_n_*-type (2 < *n* < 10) and GF*_n_*-type (2 < *n* < 9) oligosaccharides since they are obtained from hydrolysis of native inulin. Both mixtures contain smaller non-prebiotic carbohydrates such as fructose, glucose, and sucrose, resulting from the hydrolysis of the oligosaccharides and also from the enzymatic reaction itself, in the case of the microbial-FOS.

The high-length oligosaccharides present on the microbial-FOS fermentation sample seem to be preferentially consumed by the 7SC (GF_4_ > GF_3_ > GF_2_). In contrast, the preferred sugar consumption pattern for 9SC was the short-length oligosaccharide (GF_2_). Nonetheless, both bacterial consortiums seem to preferentially consume the carbohydrate with lower DP (GF_2_) on the Frutalose^®^ OFP sample. According to some studies in the literature, each bacterial strain may have specific selectivity for different FOS types and chain lengths. For instance, Kaplan and Hutkins, 2000 [47], reported that *Lactobacillus plantarum* preferentially consumed GF_2_ and GF_3_, whereas Endo et al., 2012 [39], found that main tested *Lactobacillus* strains, such as *Lactobacillus salivarius* 11741, *Lactobacillus casei* 1134, *Lactobacillus gasseri* 1131, and *Lactobacillus rhamnosus* 1136, only consumed GF_2_. While *Lactobacillus acidophilus* and all the *Bacteroides* strains tested fermented preferably GF_2_ and GF_3_. No information was provided regarding GF_4_ consumption [41]. Sonnenburg et al., 2010, found out that *Bacteroides* preferably use long-chain oligosaccharides [48]. Van Meulen et al., 2006, observed that *Bifidobacterium longum* BB536 preferentially metabolized short-chain oligofructose fractions, namely F_2_ (inulobiose), and F_3_ [49]. However, the long-chain oligofructose fractions (F_4_, GF_4_, F_5_, GF_5_, and F_6_) were also metabolized after the short-chain oligofructose fractions depletion. Regarding the *Bacteroides* spp., all oligofructose fractions were metabolized simultaneously. This suggests different oligofructose breakdown mechanisms from the *Bifidobacterium* strain and *Bacteroides* spp., which helps to explain the bifidogenic nature of inulin-type fructans. In other studies, six *Bifidobacterium* strains (*B. longum* ATCC 15707, *Bifidobacterium catenulatum* ATCC 27539, *B. adolescentis* ATCC 15705, *Bifidobacterium pseudocatenulatum* ATCC 27919, and *Bifidobacterium breve* T104) consumed both GF_2_ and GF_3_, whilst the *Bifidobacterium bifidum* JCM1254 only consumed GF_2_ [50,51]. Other studies reported that most *Bifidobacterium* strains can consume short-chain FOS (low DP) as a substrate, but not highly polymerized inulin [52,53]. Furthermore, according to Hernot et al.’s 2009 study, short-chain fructans promote bifidobacteria population growth to a higher extent than long-chain fructans [54].

Our results showed a different consumption profile between the two consortia, even with the same carbon source. This can be explained by the microorganism composition of each consortium which has specific enzyme complexes and by the cross-feeding interactions that occurred between the different microorganisms and the substrates. Both consortia had the same GF_2_ consumption rate when Frutalose^®^ OFP was used as carbon source. However, the 9SC exhibited higher GF_3_ and GF_5_ consumption rates than the 7SC. This trend can be explained by the inclusion of *B. thetaiotaomicron* and *R. faecis* in the 9SC. As previously highlighted, prior studies have reported that *B. thetaiotaomicron* preferentially consumed GF_2_ and GF_3_, which could explain the higher consumption rate of GF_3_ in the 9SC [41]. This can also explain the fact that when the microbial-FOS was used as a carbon source, the preferred sugar consumption pattern for 9SC was also the GF_2_.

Regarding metabolite production, the production of acetate in the presence of microbial-FOS and Frutalose^®^ OFP can be associated with enteric species, namely *Bacteroides*, *Lactobacillus*, and *Bifidobacterium* [55], which were present in the two studied bacterial consortia. However, it is important to note that the 9SC was composed of three species of *Bacteroides* while the 7SC is composed only of two *Bacteroides* strains (Table 1). Therefore, the 7SC consortium had one less bacterium capable of producing acetate. Acetate is the most abundant SCFA in the human gut and is also produced from acetyl-CoA derived from glycolysis and can also be transformed into butyrate by the enzyme butyryl-CoA:acetyl-CoA transferase [33]. Moreover, acetate is easily absorbed and transported to the liver [21] and is the primary substrate for cholesterol synthesis [56]. Acetate also represents a minor energy source for the colon epithelial cells.

The higher propionate production of the 9SC was expected since the main propionate-producing bacteria in the human gut belong to the genus *Bacteroides* [20], and the 9SC was composed of three species of *Bacteroides*: *B. dorei*, *B. vulgatus*, and *B. thetaiotaomicron*, while the 7SC only included the first two species. Propionate represents a minor energy source for colonocytes, but its anti-inflammatory effect is widely recognized [20].

The production of butyrate was almost absent in the 7SC fermentation because of the lack of a butyrate-producing bacteria. Meanwhile, 9SC included R. faecis, an important butyrate producer [57]. Butyrate holds an important role in human health since it is a primary energy source for colonocytes, and it has also been related to anti-inflammatory and anti-carcinogenic properties [33]. Among the substrates tested, the microbial-FOS exhibited the greatest butyrogenic effect. This observation relates to our previous findings where the butyrogenic effect was also observed in human fecal microbiota fermentations with microbial-FOS [26].

Lactate was the main organic acid produced for all the conditions tested. The lactate production is associated with the Lactobacillus species, which belong to the Firmicutes phylum [33], and in our study, both bacterial consortiums (7SC and 9SC) are composed of *L. acidophilus* and *L. rhamnosus*. It is also important to mention that lactate is considered the most common short-chain hydroxy–fatty acid in the human gut and can be converted to acetate, propionate, and butyrate through cross-feeding interactions [32,58].

According to Table 2, it is possible to observe that fermentation samples had a similar composition regarding SCFA concentration (except for lactate concentration), which was 2 times higher in the sample produced by 7SC in the presence of microbial-FOS as compared to Frutalose^®^ OFP produced metabolites. Conversely, the presence of the commercial prebiotic led to a lactate concentration increase of 1.3 times in comparison to the microbial-FOS. However, the differences observed do not fully explain the cellular metabolic activity results for the maximum concentration tested, as no significant difference was observed regarding the metabolic activity upon contact with metabolites resulting from microbial-FOS or Frutalose^®^ OFP fermentation by the 7SC. A similar result was observed for the microbial-FOS and Frutalose^®^ OFP fermentation samples of the 9SC (Figure 6). In accordance with the literature, the lactate’s role on cellular metabolic activity is still very controversial. Matsuki and colleagues [59] stated an arrest of intestinal cells in the presence of lactate and acetate; however, other authors mentioned that lactate induces intestinal cells proliferation [60]. It is important to mention that the distinct results observed in the literature may be due to the use of distinct cellular models, leading to the results’ variability. It is our understanding that due to the complexity of the obtained fermentation samples; it is not possible to clearly identify the signaling molecules responsible for the metabolic behavior observed. The decrease in the metabolite’s concentration led to an increase in the metabolic activity of our cell co-culture model being this effect significantly more pronounced on the more diluted samples (1:11) tested. Similar results were obtained in a previous work by our team using samples from fecal fermentation [61,62], where a cellular dose-dependent response behavior was observed. Another important SCFA present in fermentation samples was butyrate (Table 2). Most studies indicate a beneficial effect of butyrate on cellular metabolic activity, even though more studies are required to understand the mechanisms behind it [63]. Thus, it is necessary to conduct more cellular assays to elucidate the mechanisms behind the cellular effects observed, particularly using even more complex consortia.

## 5. Conclusions

Our work provided insight into the effects of prebiotics in two complex synthetic consortia of gut microbes. Here we show, for the first time, that the use of microbial-FOS produced by a co-culture of *A. ibericus* plus *S. cerevisiae* is a viable strategy to promote the growth of beneficial bacteria and suppress *E. coli*. This was demonstrated by the decrease in the pH caused by the production of organic acids, as well as the bacteriocins produced by lactobacilli which contributed to the inhibition of harmful bacteria growth. As a response to the addition of *B. thetaiotaomicron* and *R. faecis* to the 7SC, a change in the FOS-length bacterial consumption preference occurred, maximizing short-length oligosaccharide consumption. The profile of organic acids produced by 7SC and 9SC were also different due to the cross-feeding interactions occurring between the different bacteria. The highest production of organic acids was obtained for the 9SC, with the absence of butyrate in the 7SC being notorious. The production of SCFA was mainly promoted by the microbial-FOS, followed by Frutalose^®^ OFP in the 9SC.

Moreover, this study showed the importance of including representative bacteria of the human gut microbiota when developing an in vitro gut microbiota consortium, especially the presence of butyrate-producing species. In fact, it was observed that the 9SC bacterial metabolites promoted higher metabolic activity in cell co-culture than the 7SC (dose-dependent effect), which may be caused by the presence of butyrate, playing a protective role of the intestinal epithelium.

In conclusion, the microbial-FOS produced by our group seems to be a promising prebiotic for maintaining a balanced gut microbiota.

## Figures and Tables

**Table 1:** Bacterial culture media and growing conditions.

Bacterial Strain	Culture Media *
*Bacteroides dorei* DSM 17855	BHIS
*Bacteroides vulgatus* DSM 1447	
*Bacteroides thetaiotaomicron* DSM 2079	
*Bifidobacterium adolescentis* CECT 5781	BSM
*Bifidobacterium longum* DSM 20219	
*Lactobacillus acidophilus* CECT 288	MRS
*Lactobacillus rhamnosus* ATCC 4356	
*Roseburia faecis* DSM 16840	RBM:330
*Escherichia coli* CECT 736	NB

* BHIS—Brain Heart Infusion Broth; BSM—Bifidobacterium Selective Medium; MRS—De Man, Rogosa and Sharpe Broth; RBM:330—Rumen Bacteria Medium; NB—Nutrient Broth.

**Table 2:** Individual and total short-chain fatty acids (SCFAs) content produced during bacterial fermentations consortia with seven (7SC) or nine microorganisms (9SC) using different carbohydrate sources.

SCFA (g·L^−1^)	Time (h)	Glucose		Microbial-FOS		Frutalose^®^ OFP		Negative Control ^1^	
		7SC	9SC	7SC	9SC	7SC	9SC	7SC	9SC
Acetate	0	0.31 ± 0.00 ^a^	0.49 ± 0.01 ^b*^	0.31 ± 0.00 ^a^	0.49 ± 0.01 ^b*^	0.31 ± 0.00 ^a^	0.49 ± 0.01 ^b*^	0.31 ± 0.00 ^a^	0.49 ± 0.01 ^b*^
	12	0.81 ± 0.03 ^b^	0.83 ± 0.02 ^a^	0.66 ± 0.03 ^a^	0.97 ± 0.04 ^b^	0.72 ± 0.01 ^a^	2.30 ± 0.02 ^d*^	0.98 ± 0.02 ^c^	1.63 ± 0.05 ^c*^
	30	0.84 ± 0.01 ^a^	1.04 ± 0.05 ^a^	1.20 ± 0.01 ^b^	1.01 ± 0.10 ^a^	0.81 ± 0.01 ^a^	2.59 ± 0.08 ^c*^	0.89 ± 0.04 ^a^	1.36 ± 0.04 ^b*^
Propionate	0	1.98 ± 0.06 ^a^	2.68 ± 0.07 ^b*^	1.98 ± 0.06 ^a^	2.68 ± 0.07 ^b*^	1.98 ± 0.06 ^a^	2.68 ± 0.07 ^b*^	1.98 ± 0.06 ^a^	2.68 ± 0.07 ^b*^
	12	2.93 ± 0.04 ^c^	3.82 ± 0.05 ^c*^	2.66 ± 0.06 ^b^	3.92 ± 0.13 ^c*^	2.44 ± 0.02 ^a^	3.31 ± 0.06 ^b*^	2.65 ± 0.03 ^b^	2.81 ± 0.04 ^a*^
	30	3.05 ± 0.05 ^d^	3.81 ± 0.14 ^b*^	2.77 ± 0.05 ^c^	3.85 ± 0.11 ^b*^	2.48 ± 0.00 ^b^	2.84 ± 0.08 ^a*^	1.96 ± 0.03 ^a^	3.15 ± 0.07 ^a*^
Butyrate	0	0.12 ± 0.00 ^a^	1.17 ± 0.02 ^b*^	0.12 ± 0.00 ^a^	1.17 ± 0.02 ^b*^	0.12 ± 0.00 ^a^	1.17 ± 0.02 ^b*^	0.12 ± 0.00 ^a^	1.17 ± 0.02 ^b*^
	12	0.18 ± 0.00 ^c^	1.58 ± 0.14 ^a*^	0.13 ± 0.01 ^b^	2.71 ± 0.07 ^c*^	0.18 ± 0.00 ^c^	1.88 ± 0.03 ^b*^	0.07 ± 0.00 ^a^	1.33 ± 0.05 ^a*^
	30	0.18 ± 0.00 ^c^	1.66 ± 0.08 ^a*^	0.13 ± 0.00 ^b^	2.97 ± 0.06 ^c*^	0.18 ± 0.00 ^c^	2.26 ± 0.03 ^b*^	0.07 ± 0.00 ^a^	1.52 ± 0.06 ^a*^
Lactate	0	4.43 ± 0.07 ^a^	4.71 ± 0.13 ^a^	4.43 ± 0.07 ^a^	4.71 ± 0.13 ^a^	4.43 ± 0.07 ^a^	4.71 ± 0.13 ^a^	4.43 ± 0.07 ^a^	4.71 ± 0.13 ^a^
	12	7.71 ± 0.04 ^d^	10.59 ± 0.15 ^c*^	6.72 ± 0.07 ^c^	8.94 ± 0.23 ^b*^	4.53 ± 0.04 ^b^	14.00 ± 0.18 ^d*^	3.60 ± 0.04 ^a^	5.20 ± 0.05 ^a*^
	30	9.87 ± 0.08 ^c^	15.11 ± 0.13 ^d*^	12.17 ± 0.01 ^d^	10.88 ± 0.20 ^b*^	5.93 ± 0.01 ^b^	14.07 ± 0.11 ^c*^	3.73 ± 0.04 ^a^	8.47 ± 0.16 ^a*^
Total SCFA	0	2.41 ± 0.06 ^a^	4.34 ± 0.09 ^b*^	2.41 ± 0.06 ^a^	4.34 ± 0.09 ^b*^	2.41 ± 0.06 ^a^	4.34 ± 0.09 ^b*^	2.41 ± 0.06 ^a^	4.34 ± 0.09 ^b*^
	12	3.91 ± 0.07 ^c^	6.23 ± 0.14 ^a*^	3.45 ± 0.08 ^a^	7.60 ± 0.23 ^b*^	3.34 ± 0.02 ^a^	7.48 ± 0.07 ^b*^	3.70 ± 0.05 ^b^	5.77 ± 0.14 ^a*^
	30	4.08 ± 0.06 ^c^	6.51 ± 0.23 ^a*^	4.11 ± 0.04 ^c^	7.83 ± 0.20 ^b*^	3.48 ± 0.01 ^b^	7.70 ± 0.13 ^b*^	2.92 ± 0.03 ^a^	6.03 ± 0.13 ^a*^

^1^ Negative control: no-added carbohydrates to fermentation media. Values are presented in g·L^−1^ ± SD. Different superscript letters correspond to significant differences (*p* < 0.05) between treatments of the same bacterial consortium and time. Superscript asterisks correspond to significant differences (*p* < 0.05) among bacterial consortia for the same treatment and time.

## Data Availability

The data presented in this study are available on request from the corresponding author.
